# Rutin–Whey Protein Nanoparticles Inhibit D-Galactose-Induced Skeletal Muscle Dysfunction by Modulating Gut Microbiota and Metabolic Pathways

**DOI:** 10.3390/nu17101734

**Published:** 2025-05-20

**Authors:** Yijing Ren, Lianyan Wang, Danyang Wang, Jian Huang, Ou Wang, Gangqiang Ding

**Affiliations:** 1 School of Food and Health, Beijing Technology and Business University, Beijing 100048, China; ryjing3021@163.com; 2 NHC Key Laboratory of Public Nutrition and Health, National Institute for Nutrition and Health, Chinese Center for Disease Control and Prevention, Beijing 100050, China; huangjian@ninh.chinacdc.cn; 3 Institute of Process Engineering, Chinese Academy of Sciences, Beijing 100190, China; wanglianyan@ipe.ac.cn (L.W.); wangdanyang24@ipe.ac.cn (D.W.); 4 University of Chinese Academy of Sciences, Beijing 100190, China

**Keywords:** rutin, whey protein, delivery system, muscle strength

## Abstract

**Background**: Rutin (R) is a bioactive compound with antioxidant and anti-inflammatory properties, but its low bioavailability limits its application. To address this problem, R was encapsulated with whey protein (W) as nanoparticles, and the potential effect and mechanism of rutin–whey protein nanoparticles (RW) on skeletal muscle dysfunction was investigated in D-galactose induced mice. **Methods**: R was encapsulated with W to form RW, and its characteristics like particle size, encapsulation efficiency, and bioaccessibility were evaluated. In the in vivo study, male C57BL/6J mice were treated with R, W or RW, respectively. The muscle function, hepatic antioxidant capacity, serum inflammatory levels, gut microbiota, and metabolomic profiles of mice were evaluated. **Results**: RW showed a uniform particle size, with an encapsulation efficiency of 68.7%. In the RW, the bioaccessibility of rutin was approximately 3.3 times that of free rutin. This in vivo study indicated that in comparison with D-galactose induced mice (model group), R, W and RW treatments could enhance hepatic antioxidative capacity and regulate inflammation levels, while W and RW could also increase muscle strength. Among these, RW treatment significantly elevated the hepatic GSH-PX activity and decreased the serum MSTN, TNF-α, and IL-6 levels, which were all markedly better than those of the individual effect of R or W. Such effects of R, W, and RW may be achieved through the modulation of gut microbiota that produced short-chain fatty acids or involved in anti-inflammatory function and the regulation of metabolic profiles associated with amino acid metabolism, aminoacyl-tRNA biosynthesis, etc. **Conclusions**: RW was found to enhance the bioaccessibility of rutin, and exhibited positive effects on skeletal muscle dysfunction via the modulation of gut microbiota and metabolic pathways. The results of this study may provide new scientific strategy for the utilization of rutin to achieve its health benefits.

## 1. Introduction

Rutin, also known as rutoside, is a dietary flavonoid that is widely distributed in vegetables and fruits [1]. It exhibits a variety of healthy effects, such as antioxidant, anti-allergy, anti-cancer, etc. [2]. Due to its health advantages, there has been increasing interest in rutin application in pharmacy and functional food development [3]. However, such utilization is somewhat limited by the low solubility and poor bioavailability of rutin [4]. According to published reports, rutin is a highly hydrophobic molecule with a water solubility of 0.13 g/L [5], and the oral bioavailability of it is of less than 5% in SD rats [6], which hinders processing and efficient application.

The development of a food delivery system presented a viable strategy to overcome the challenges associated with rutin application. Research indicates that rutin could be effectively encapsulated into nanoparticles [7], liposome [8], or nanoemulsions [9] to enhance its bioavailability and functionality. For example, in comparison with rutin in free form, the oral bioavailability of chitosan-coated rutin nanoparticles was increased by 4.2 folds [7]. Similarly, the bioaccessibility of rutin in the form of a Pickering emulsion exhibited a two-fold increase [9].

Among the carriers of the delivery system, whey protein is a kind of milk-derived protein, and it shows the abilities of binding with hydrophobic bioactive compounds, gel formation, and emulsification [10]. As published studies reported, whey protein could encapsulate phenolic compounds such as rutin and chlorogenic acid from *Lycium barbarum* leaf extract into nanoparticles, and the bioaccessibility of the total phenolics in *Lycium barbarum* leaf extract was increased by 53.7% after simulated digestion in vitro [11]. Additionally, the bioavailability of free pterostilbene was of less than 3%; however, when encapsulated in whey protein as nanoparticles, it could be significantly increased to 44.7%, and whey protein could also protect the pterostilbene from loss in the gastrointestinal environment [12].

In addition to the potential for delivery system construction, whey protein also has high nutritional value for muscle health. Whey protein is characterized by its abundant branched-chain amino acid content, particularly leucine, whose utility as a dietary supplement for improving muscular strength or mass and mitigating muscle atrophy has been established [13,14]. For example, leucine could stimulate muscle protein synthesis through the activation of the mammalian target of rapamycin (mTOR) signaling cascade, which is a key signaling pathway regulating protein synthesis [15]. In addition, the acute supplementation of leucine or whey protein could restore the stimulation of muscle protein synthesis in aged rats and seniors [16,17].

Moreover, polyphenols are natural phytochemicals with outstanding antioxidant properties, and they also exhibit benefits for muscle health. In the published studies, curcumin could attenuate muscle protein breakdown by activating histone deacetylase sirtuin-1, which lessened muscular atrophy in mice [18]; quercetin exhibited protective effects against obesity-associated skeletal muscle atrophy by inhibiting the inflammatory receptor Toll-like receptor 4 (TLR4) in mice [19]. Therefore, as a natural antioxidant, rutin may also be a potential agent for muscle health protection.

Based on these, rutin and whey protein are both food-derived components that exhibit potential benefits on skeletal muscle health. There is also a possibility to establish the delivery system between these. Therefore, the aim of this study was to develop a rutin–whey protein nanoparticle (RW) to improve the bioavailability of rutin, and to evaluate its positive effects and potential mechanism on muscle function protection in mice with skeletal muscle dysfunction.

## 2. Materials and Methods

### 2.1. Reagents and Chemicals

Rutin (98%) was sourced from Nanjing Jingzhu Biotechnology Co., Ltd. (Nanjing, China). Whey protein (98%) was acquired from DAVISCO, Savage, MN, USA. D-galactose (D-gal) was purchased from Sigma Aldrich (St Louis, MO, USA). Pig bile salts were purchased from Shanghai Yuan Ye Biotechnology Co., Ltd. (Shanghai, China). Pepsin was obtained from Shanghai Mecklin Biochemical Technology Co., Ltd. (Shanghai, China). Trypsin was purchased from Shanghai Yien Chemical Technology Co., Ltd. (Shanghai, China). A bicinchoninic acid (BCA) protein assay kit was purchased from JinClone Biotechnology Co., Ltd. (Beijing, China). Malondialdehyde (MDA), catalase (CAT) and glutathione peroxidase (GSH-PX) assay kits were purchased from Nanjing Jiancheng Bioengineering Research Institute Co., Ltd. (Nanjing, China). The mouse tumor necrosis factor alpha (TNF-α), mouse interleukin-6 (IL-6) and mouse muscle growth inhibitor (MSTN) ELISA kits were purchased from Enzyme Label Biotechnology (Yancheng, China). The chemicals used for metabolomics analysis, including chromatographic-grade methanol, chromatographic-grade acetonitrile, chromatographic-grade formic acid, chromatographic-grade propanol, and chromatographic-grade water, were purchased from Fisher Scientific (Shanghai, China). Chromatographic-grade chloroform was obtained from Vokai Biotechnology Co. (Beijing, China), chromatographic-grade pyridine from Aladdin (Shanghai, China), methoxyamine hydrochloride and L-2-chlorophenylalanine from Damas-beta (Shanghai, China), and Bis(trimethylsilyl)trifluoroacetamide (BSTFA) (1% Chlorotrimethylsilane (TMCS)) from Regis (Shanghai, China). The other reagents were analytically pure.

### 2.2. Preparation and Characteristics of Rutin–Whey Protein Nanoparticles

**Figure 1:**
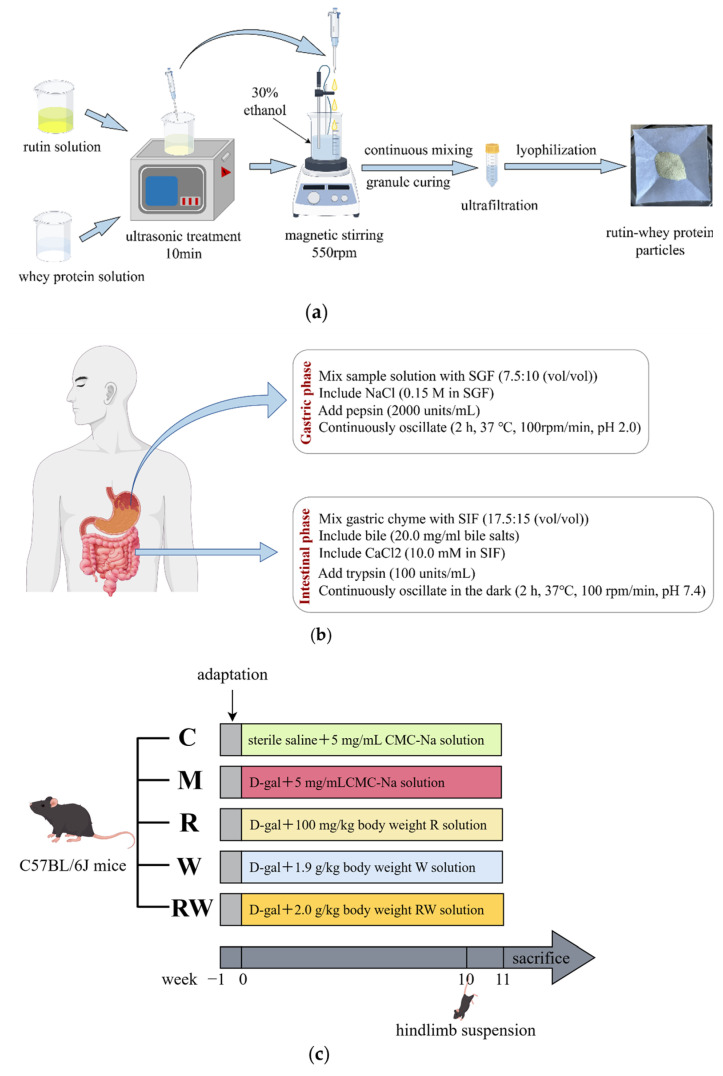
Flow chart of the in vitro and in vivo study method (by Figdraw). (**a**) Preparation flowchart of RW. (**b**) Flowchart of in vitro digestion. (**c**) Animal experiment design. C was the control group, M was the model group, R was the rutin group, W was the whey protein group, RW was the rutin–whey protein nanoparticle group.

Rutin was dissolved in a 1 M NaOH solution at a concentration of 4.0 mg/mL and mixed with 8% whey protein solution at a volume ratio of 1:1. Then, the mixture was ultrasonicated at 90% amplitude for 10 min and dripped into a 30% ethanol solution under 550 rpm magnetic stirring. The whole solution system was continuously stirred for 3 h until the nanoparticles were fully solidified. Subsequently, the nanoparticles were separated from the solution via centrifugation using a 100 kD ultrafiltration tube at 5000 rpm for 15 min, followed by lyophilization as RW (Figure 1a). The lower layer of the centrifuged solution in the ultrafiltration tube was collected, absorbance was measured at 396 nm, and the concentration of unencapsulated rutin was calculated. The encapsulation efficiency and loading capacity of the nanocarriers were calculated as shown in (1) and (2):

Encapsulation Efficiency = (m_total rutin_ − m_free rutin_)/m_total rutin_ × 100% (1)

Loading Capacity = (m_total rutin_ − m_free rutin_)/m_total nanospheres_ (2)

In the formulas, m_total rutin_ indicates total rutin (μg), m_free rutin_ indicates unencapsulated rutin (μg), and m_total nanospheres_ indicates total whey protein nanospheres (mg).

The particle size and size distribution profiles of RW were characterized using dynamic light-scattering analysis. Briefly, RW was suspended and diluted, and then transferred to the sample cell. Once stabilized, the particle size and size distribution of RW were determined at 25 °C. The uniformity of the particle size was evaluated by the polydispersity index (PDI). The experimental procedures for preparing and characterizing RW were conducted in triplicate.

### 2.3. In Vitro Digestion of RW

Simulated gastrointestinal digestion was performed according to previous studies with minor modifications (Figure 1b) [20,21]. The simulated gastric fluid (SGF) was prepared as 0.15 mol/L NaCl and 2000 units /mL pepsin, with the pH adjusted to 2.0. Similarly, the simulated intestinal fluid (SIF) was formulated with 10.0 mmol/L CaCl_2_, 20.0 mg/mL bile salts, and 100 units/mL trypsin, and the pH value was 7.4. For the simulated digestion, RW was suspended in double-distilled water at a concentration of 5.0 mg/mL, 7.5 mL of it was mixed with 10.0 mL SGF, and the pH value was set to 2.0. Then, the mixture was continuously oscillated at 100 rpm/min and 37 °C for 2 h to simulate gastric digestion. After this, about 15.0 mL SIF was added to the mixture and the pH value was adjusted to 7.4. Subsequently, the whole system underwent continuous shaking at 100 rpm/min and 37 °C in the dark. About two hours later, the digest was collected and centrifuged at 10,000× *g* for 30 min, and the supernatant was collected as a mixed micellar phase. Rutin was extracted from the micellar phase with dimethyl sulfoxide, and absorbance was read at 364 nm. The retention rate and bioaccessibility of rutin were calculated as shown in (3) and (4):

Retention rate (%) = M_t_/M_0_ × 100% (3)

Bioaccessibility (%) = M_micelle_/M_0_ × 100% (4)

In the equations, M_t_ indicates the residual content of rutin in the solutions, M_0_ indicates the initial content of rutin in the initial solution, and M_micelle_ indicates the rutin content in the micellar phase.

### 2.4. Mice Experiment

#### 2.4.1. Animals and Experimental Design

Forty-five eight-week-old male C57BL/6J mice weighing 22.5 ± 2 g were purchased from Beijing Viton Lever Laboratory Animal Technology Co., Ltd. (Beijing, China; SCXK (Jing) 2016-0006), and housed under conditions of a 12 h light–dark cycle and a temperature of 24 ± 1 °C. The cage size was 320 mm × 215 mm × 170 mm, made of plastic, and lined with sterile corn cob bedding during the experiment. The number of mice housed in each cage was less than five. During the study, the animals had free access to food and water, and after a seven-day adaptation, they were randomly divided into five groups (*n*=9) based on body weight: the control group (C), the model group (M), the rutin group (R), the whey protein group (W), and the rutin–whey protein nanoparticle group (RW). Each group received different interventions for 11 weeks, with injections and gavage treatments, as shown in Figure 1c. D-gal was dissolved in sterile saline and intraperitoneally injected into mice at 400 mg/kg of body weight to induce muscle dysfunction, except in the C group. R, W, and RW were suspended with 5 mg/mL solution of carboxymethyl cellulose sodium (CMC-Na) for gavage administration. Gavage and injection were administered once per day for seven days per week, and the volume of injection and gavage was adjusted by weekly body weight. Hindlimb suspension was initiated in the 10th week of the experiment along with continuous gavage and injection treatments, which can simulate a weightless condition, reduce the use of skeletal muscles, and promote muscle atrophy [22]. As published studies indicate, weight loss, decreased muscular endurance, reduced grip strength, etc., are typical symptoms of aging [23,24]. This study was approved by the Ethics Committee of the National Institute for Nutrition and Health, China CDC (approval No. 2023-NINH-MOD-001).

#### 2.4.2. Skeletal Muscle Movement Ability and Grip Strength Test

At the 11th week of the experiment, the muscle movement endurance of mice was evaluated with a small animal treadmill (ZS-PT-III, Beijing Zhongshidichuang Technology Development Co., Beijing, China). The forelimb grip strength of mice was tested with a grip strength meter (ZS-ZL, Beijing Zhongshidichuang Technology Development Co.). The testing methods can be found in the Supplementary Materials.

#### 2.4.3. Sample Collection and Analysis

During the final experimental week, fresh fecal samples were individually collected from mice using sterile tubes and immediately stored at −80 °C for subsequent microbiota profiling. The collection method was depicted in the Supplementary Materials. At the end of the intervention, the mice were fasted overnight. Blood was collected from the orbital plexus and the serum was separated by centrifugation at 3500× *g* for 15 min at 4 °C. Mice were exsanguinated after anesthesia with chloral hydrate solution. After sacrificing, the liver and both sides of the gastrocnemius muscle were collected separately. The liver and one side of the gastrocnemius muscle were placed in liquid nitrogen and subsequently stored at −80 °C. The other side of the gastrocnemius muscle was fixed in tissue-fixative solution for histological analysis.

For the biochemical analysis, the liver was homogenized on ice. The hepatic MDA content, CAT activity, GSH-PX activity and serum MSTN, TNF-α and IL-6 levels were measured according to the commercial assay kit’s instructions.

For the histologic observation, the gastrocnemius muscle was embedded in paraffin, and the slices were stained with hematoxylin and eosin (HE) for observation.

#### 2.4.4. Gut Microbiota Analysis

DNA was extracted from feces and 16S rRNA gene sequencing was conducted following validated protocols, as indicated by [25]. Briefly, total microbial genomic DNA was extracted from fecal samples according to the kit’s instructions. The quantification and purity assessment of DNA samples were conducted using agarose gel electrophoresis (1.0% concentration) and the NanoDrop2000 instrument (Thermo Scientific Inc., Waltham, MA, USA), respectively. The hypervariable regions V3-V4 of the 16S rRNA gene were amplified using 338F and 806R primers. The amplified PCR product was extracted and purified from a 2% agarose gel. Quantification was performed by Synergy HTX (Biotek, Winooski, VT, USA). High-throughput sequencing was performed by Majorbio Bio-Pharm Technology Co., Ltd. (Shanghai, China). The data analysis method is provided in the Supplementary Materials.

#### 2.4.5. Metabolomics Analysis

Briefly, 0.5 mL of a methanol–water solution (4:1, *v*/*v*), 200 µL of chloroform, and 50 mg of gastrocnemius muscle were added to the grinding tube. The internal standard was a 0.05 mg/mL ribitol solution. The mixture was frozen and grounded, ultrasonicated, and centrifuged to collect supernatant. The supernatant was blown to dry with nitrogen. Then, an 80 μL aliquot of a 15 mg/mL methoxypyridine hydrochloride solution was introduced. Following 2 min vortex mixing, an oximation reaction was carried out in a shaking incubator at 37 °C for 90 min. Subsequently, 80 μL of BSTFA derivatization reagent, which contained 1% TMCS, was added. The reaction mixture was vortexed and shaken for 2 min and incubated at 70 °C for 60 min. Finally, the samples were left at ambient room temperature for 30 min. The metabolomics analysis was performed at Majorbio Biomedical Technologies using Agilent 8890B gas chromatography and a 5977B mass selective detector (Shanghai, China). The detailed procedures, conditions, and data analysis are provided in the Supplementary Materials.

### 2.5. Data Analysis

Data were expressed as mean ± standard error and analyzed with SPSS software (Version 22.0; IBM Inc., Chicago, IL, USA). The results of the in vitro experiments were analyzed by independent samples’ *t*-tests. The results of the in vivo experiments were analyzed with one-way ANOVA, followed by Tukey’s test. A *p*-value of less than 0.05 was considered significantly different.

## 3. Results and Discussion

### 3.1. Characteristics of RW

**Figure 2:**
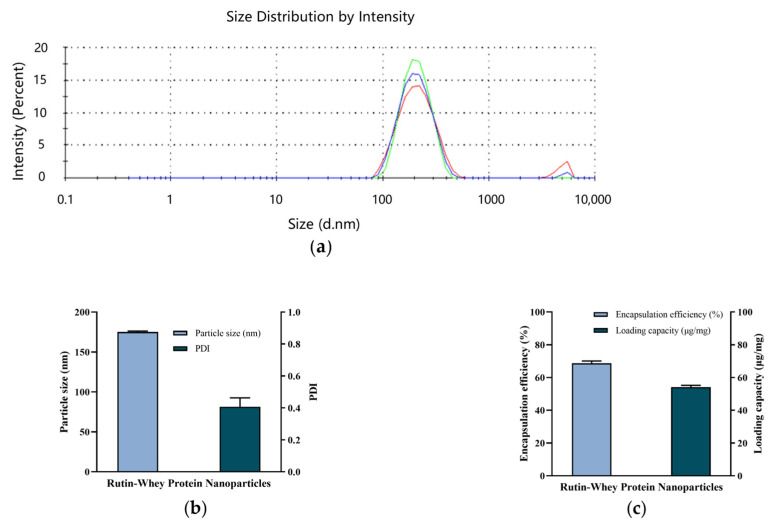
Characteristics of RW. (**a**) Particle size distribution. Lines with different colors represent different repeats. (**b**) Particle size and PDI. (**c**) Encapsulation efficiency and loading capacity. Data are expressed as mean ± S.E. (*n* = 3).

As illustrated in Figure 2a,b, the particle size of RW was uniform, the PDI was 0.407 ± 0.056, and the average particle diameter was 175.2 ± 1.1 nm. In previously published studies, rutin could be encapsulated in quinoa starch and corn starch with encapsulation efficiencies of 67.4% and 63.1%, respectively, along with loading efficiencies of 26.6% and 22.7%, respectively [26]. When combined with whey protein, in this study, the rutin encapsulation efficiency was about 68.7 ± 1.3%, and the loading amount was about 54.1 ± 1.1 μg/mg nanoparticles (Figure 2c).

### 3.2. In Vitro Digestion and Bioaccessibility

**Figure 3:**
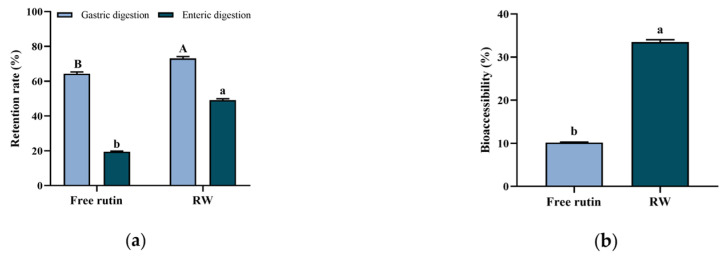
In vitro digestion of free rutin and RW. (**a**) Retention rate of free rutin and RW. (**b**) Bioaccessibility of free rutin and RW. Data are expressed as mean ± S.E. (*n* = 3). Different letters indicate significant differences (*p* < 0.05). In Figure (**a**) uppercase letters indicate differences in gastric digestion and lowercase letters indicate differences in enteric digestion.

Bioactive compounds, such as rutin, are easy to be degraded or biotransformed by enzymes in the gastrointestinal system, and lead to absorption difficulty or bioavailability decrease [27]. Such application problems could be addressed by edible delivery systems’ construction. In this study, whey protein was used to encapsulate with rutin as a nanoparticle, and the bioaccessibility of RW was investigated by in vitro digestion simulation. As shown in Figure 3a, after stomach and intestinal digestion, the free rutin retention rate was 19.5 ± 0.3%, while the retention rate of rutin in RW was 49.2 ± 0.7%, which showed a statistically significant difference (*p* < 0.05).

Bioaccessibility can be defined as the ability of the compound to be released from the food matrix and be available for intestinal absorption after digestion [28]. As previous findings reported, protein in the delivery systems exhibited potential to enhance the bioaccessibility of bioactive compounds. For example, soy isoflavones could be encapsulated in whey protein nanoparticles and showed enhanced stability and bioaccessibility during simulated digestion [21]. Similarly, in this study, the bioaccessibility of rutin in RW was 33.5 ± 0.6%, which was approximately 3.3 times that of free rutin (*p* < 0.05, Figure 3b). A previously published study indicated that whey protein as the wall material of the delivery system may increase the dissolution of the core compound within the mixed micellar phase after simulating gastrointestinal digestion and lead to an improvement in bioaccessibility [21].

### 3.3. Effects of RW on Muscle Function, Antioxidative Capacity and Cytokine Levels in Mice

**Figure 4:**
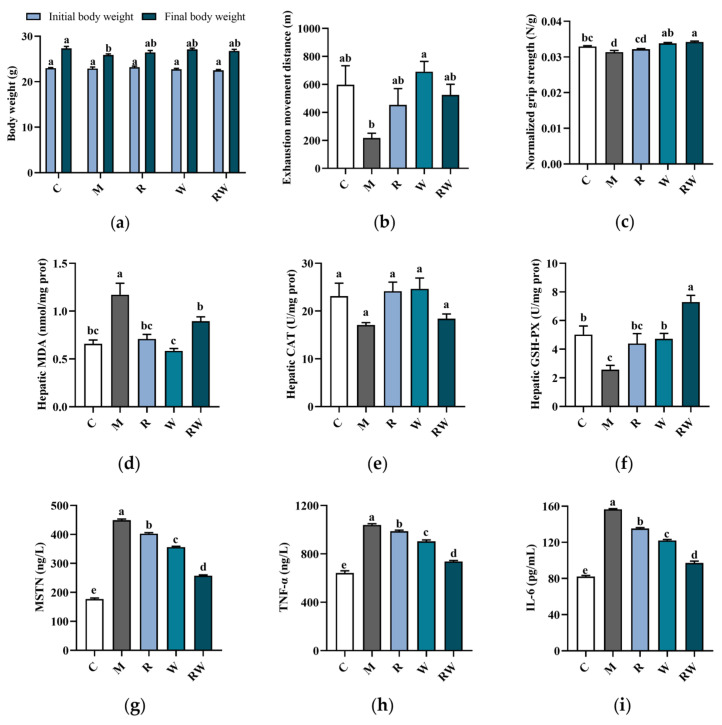
Effects of R, W and RW treatments on body weight, muscle function, antioxidative capacity and cytokine levels. (**a**) Body weight change. (**b**) Exhaustion movement distance. (**c**) Normalized grip strength. (**d**) Hepatic MDA level. (**e**) Hepatic CAT activity. (**f**) Hepatic GSH-PX activity. (**g**) Serum MSTN level. (**h**) Serum TNF-α level. (**i**) Serum IL-6 level. Data are expressed as mean ± S.E. (*n* = 6 or 8). Different letters of the same parameters indicate significant differences (*p* < 0.05). C was the control group, M was the model group, R was the rutin group, W was the whey protein group, RW was the rutin–whey protein nanoparticle group.

In this study, mice were intraperitoneally injected with D-gal for 11 weeks, along with the R, W and RW administrations. In addition, hindlimb suspension was also performed in the 10th week, which can simulate a weightless condition, reduce the use of skeletal muscles, and promote muscle atrophy [22]. At the end of the experiment, some typical aging symptoms were observed in the mice of the M group. As shown in Figure 4a–c, compared with the mice in the C group, the final body weight and grip strength of mice in the M group were significantly decreased (*p* < 0.05). The distance of exhaustion exercise of the mice in the M group was also lower than that of the C group mice, but with no statistically significant difference. Under the treatments of R, W and RW, the aging characteristics were improved to different extents. As shown in Figure 4a, the final body weight of the mice under the R, W and RW treatments showed an increasing tendency when compared with that of the mice in the M group, although without a statistically significant difference. In addition, when compared with the mice in the M group, the grip strength of the mice under W administration was markedly increased (*p* < 0.05). Rutin treatment exhibited an insignificant effect on forelimb grip strength in comparison with that of the M group mice. However, when rutin was combined with whey protein as nanoparticles, the RW administration significantly increased the grip strength by 9.19% (compared with the M group, *p* < 0.05).

Oxidative stress was typically characterized by an increase in reactive oxygen species (ROS) production, which may affect muscle function and lead to muscle atrophy and strength loss [29]. Therefore, the oxidative stress status of the mice was evaluated and compared in this study to further illustrate the potential change in muscle function. The CAT and GSH-PX were typical enzymatic antioxidants to mitigate reactive species [30], and MDA was the final product to evaluate the lipid peroxidation status [31]. As the data show in Figure 4d–f, in comparison with the mice in the C group, the mice in the M group showed a significant increase in the hepatic MDA level and a marked reduction in GSH-PX activity (*p* < 0.05), which indicated a decrease in antioxidative capability in vivo. Rutin is well known for its antioxidant ability, and whey protein could also enhance the body’s antioxidant capacity [2,32]. Consistent with previous reports, in this study, when mice were administrated R, W and RW, the hepatic MDA levels were all significantly lower than those of the M group mice (*p* < 0.05). In addition, in comparison with the mice of the M group, the W and RW interventions could also significantly elevate the GSH-PX activity, with the RW administration exhibiting a markedly better effect than the W intervention (*p* < 0.05).

The levels of inflammatory cytokines and myokines were also important indicators of skeletal muscle health. The aging process was characterized by a progressive increase in systemic inflammation, which was often accompanied by elevated levels of key pro-inflammatory cytokines, such as TNF-α and IL-6 [33]. These pro-inflammatory factors may contribute to muscle wasting by simultaneously enhancing protein degradation and suppressing muscle protein synthesis [33]. In addition, as a member of the transforming growth factor-β (TGF-β) superfamily, MSTN was predominantly expressed in skeletal muscle and exerted an inhibitory effect on muscle growth [34]. In accordance with previous reports [35,36], the serum MSTN, TNF-α and IL-6 concentrations of the mice in the M group were all significantly higher than those of the C group (Figure 4g–i, *p* < 0.05), which suggested a potential chronic inflammation under D-gal induction and may further be associated with muscle dysfunction or atrophy. When the mice were administrated R, W or RW, the serum pro-inflammatory factors and myokine levels were all markedly decreased (Figure 4g–i, *p* < 0.05, compared with the M group). Among the three intervention groups, the serum MSTN, TNF-α, and IL-6 levels under RW administration were decreased by 42.6%, 29.1%, and 37.9%, respectively, when compared with those of the M group (*p* < 0.05), which was significantly better than the individual effects of R and W (*p* < 0.05). Therefore, there may be a potential synergistic effect of rutin and whey protein on inflammatory cytokines’ regulation.

The degeneration of skeletal muscle structure is a typical manifestation of sarcopenia [37]. As shown in Figure S2 in the Supplementary Materials, the muscle tissue of the mice in the C group was connective, the muscle cells were arranged, and the muscle fibers were generally uniform, with no fiber rupture or infiltration of inflammatory cells. In comparison, the mice in the M group showed a loose arrangement of the muscle cells, the muscle fibers showed uneven sizes and ruptures, the gaps between the muscle fibers were enlarged, interstitial edema was present, and numerous inflammatory cells infiltrated between the muscle cells. These changes indicate that the mice under D-gal administration exhibited skeletal muscle structure damage. The R and W interventions showed similar slightly protective effects on muscle structure, while such a protective effect was also obvious in RW group, as fewer inflammatory cells infiltrated the muscle tissue.

### 3.4. Results of Gut Microbiota Analysis

**Figure 5:**
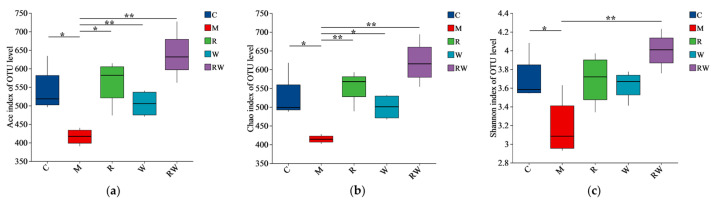

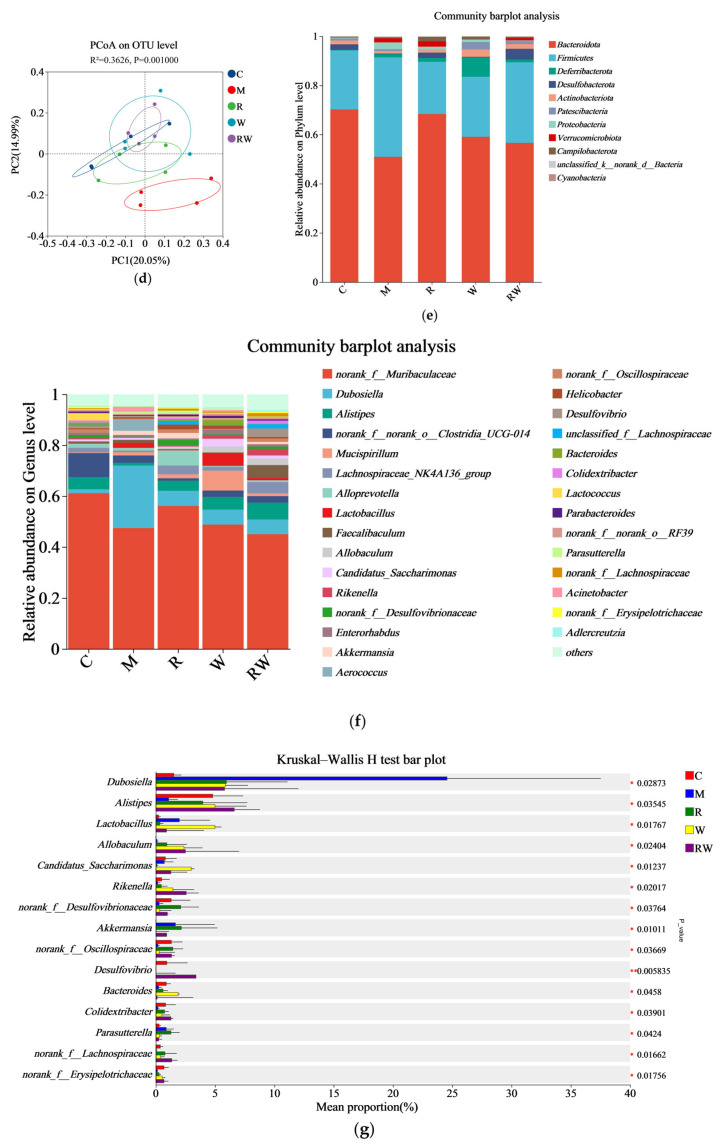

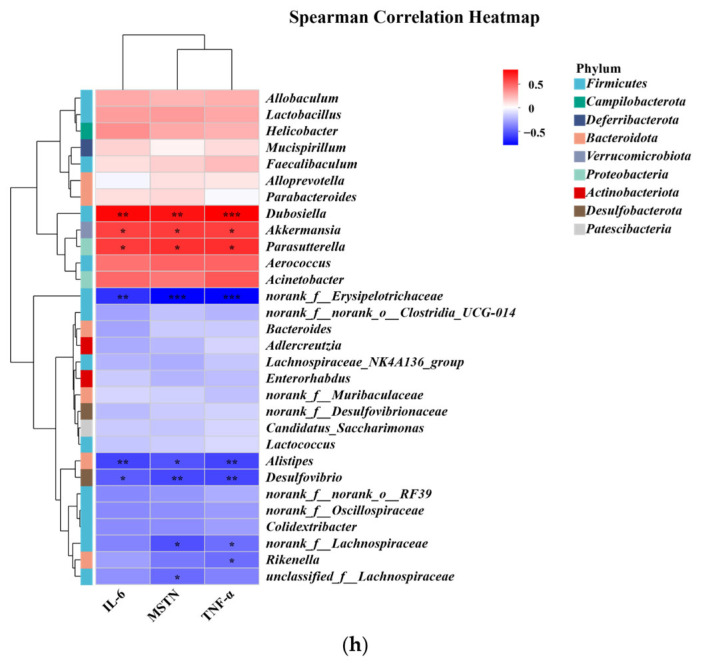
Effects of the R, W and RW treatments on the gut microbiota. (**a**) Ace index. (**b**) Chao index. (**c**) Shannon index. (**d**) Principal coordinates analysis diagram of the OTU level. (**e**) Relative abundance of microbiota at the phylum level. (**f**) Relative abundance at the genus level. (**g**) Analysis of differences in species abundance. (**h**) Spearman’s correlation analysis between inflammatory factors and gut microbiota. C was the control group, M was the model group, R was the rutin group, W was the whey protein group, RW was the rutin–whey protein nanoparticle group. * *p* < 0.05 vs. M group, ** *p* < 0.01 vs. M group. The color scale in the legend of the correlation analysis represents different R values, with red corresponding to a positive correlation and blue corresponding to a negative correlation. * *p* < 0.05, ** *p* < 0.01, *** *p* < 0.001.

The 16S rRNA sequences of mice fecal microorganisms were analyzed. As shown in the Supplementary Materials (Figure S3), the numbers of OTUs in the C, M, R, W, and RW groups were 112, 104, 114, 109, and 117, respectively, with 87 OTUs were shared. The α-diversity, often evaluated as Ace, Chao, and Shannon diversity indices, is usually used to analyze gut microbiota diversity among each group [38]. As demonstrated in Figure 5a–c, the Ace, Chao, and Shannon indices of M group were significantly lower than those of the C group (*p* < 0.05). Compared with those of the M group, the mice under the R and W interventions showed marked increases in the Ace and Chao indices, while the mice under the RW intervention showed notable increases in the Ace, Chao, and Shannon indices (*p* < 0.05). These results indicate that the mice in the M group experienced considerable alterations in bacterial diversity and species richness, which were restored by the different interventions in this study. Furthermore, a principal coordinate analysis (PCoA) of β-diversity was conducted to identify the differences in the gut flora structure among the groups. As shown in Figure 5d, the cluster of the M group was clearly distinguished from that of the other groups, while the clusters of the R, W, and RW intervention groups were close to that of the C group, which indicated that the three interventions may reverse the change in the intestinal flora structure induced by D-gal.

Evidence suggests that gut flora dysbiosis might cause an innate immunological response, chronic inflammation, and anabolic resistance, which contributes to age-related degeneration, cognitive decline, and aging [39,40]. The age-related alterations in gut microbiota composition may contribute to the development of sarcopenia through the gut–muscle axis [40]. To identify the specific alterations in the fecal flora, the changes in gut microbial composition were analyzed from the phylum and genus levels. Figure 5e shows the relative abundance of the major phyla, with *Bacteroidota* and *Firmicutes* as the dominating phyla among the five groups. The change in the ratio between *Firmicutes* and *Bacteroidota* (*F/B*) may occur with aging and then influence the health status of the host. Previous studies have shown that an increased abundance of *Firmicutes* and a reduced abundance of *Bacteroidota* lead to an elevated *F/B* ratio in senescent mice, which might be related to reduced muscle mass and function [41,42]. Similarly, in the current study, the *F*/*B* ratio of the M group was numerically higher than that of the C group, while such an increase was mitigated by the R, W, and RW treatments (Figure S4). However, such a change was not statistically different.

In this study, the relative abundance of major genera is depicted in Figure 5f, with the leading groups being *norank_f_Muribaculaceae, Dubosiella*, and *Alistipes*. At the genus level, significant differences in relative abundances between different groups were determined using the Kruskal–Wallis rank sum test. The results revealed that in the M group, the relative abundances of bacteria such as *Alistipes*, *Rikenella*, *Bacteroides*, *Colidextribacter*, *norank_f_Oscillospiraceae* and *norank_f_Lachnospiraceae* were decreased, while the relative abundance of *Parasutterella* was increased (compared with the C group, Figure 5g). The *norank_f_Oscillospiraceae* and *norank_f_Lachnospiraceae* belong to the *Oscillospiraceae* family and *Lachnospiraceae* family, respectively, and both can produce short-chain fatty acids (SCFAs) [43,44]. In addition, *Alistipes* is also an SCFA-producing microorganism [45]. In recent years, a growing amount of evidence proved that muscle function can be regulated by the gut microbiota, thus establishing the concept of the “gut–muscle axis” [46]. SCFAs are metabolic products derived from the colonic microbial fermentation of undigested starch and non-starch polysaccharides [47]. The current research demonstrated that injection of SCFAs-producing probiotics could alter the composition of the gut microbiota, concomitant with reduced systemic inflammatory markers, and increase skeletal muscle mass in mice under a high-fat diet [48]. Meanwhile, it has also been proven that low levels of SCFAs in the gut microbiota might worsen subclinical chronic inflammation and result in sarcopenia [49]. Therefore, SCFAs are one of the important mediators between the gut microbiota and muscle health. In this study, compared to the M group, the R, W, and RW interventions increased the relative abundances of *norank_f_Oscillospiraceae*, *norank_f_Lachnospiraceae* and *Alistipes* (Figure 5g). Therefore, according to the theory of the gut–muscle axis, by influencing the gut microbiota composition, the treatments of R, W and RW may further mediate SCFA production and contribute to muscle health protection.

In addition, inflammation is another important factor in the gut–muscle axis. Persistent inflammation may lead to decreased skeletal muscle mass and function [50]. *Colidextribacter* and *Rikenella* are potential anti-inflammatory probiotics that have a role in reducing intestinal inflammation [51,52,53]. Recent investigations have demonstrated a positive correlation between the abundance of *Parasutterella* and the presence of chronic intestinal inflammation in patients clinically diagnosed with inflammatory bowel disease [54]. In our study, the relative abundances of *Colidextribacter* and *Rikenella* were increased by the R, W, and RW treatments, while the W and RW administrations reduced the relative abundance of *Parasutterella* in mice, with the RW intervention exhibiting a numerically better effect than the individual administration of R or W (Figure 5g).

Spearman’s correlation analysis was further applied to elucidate the relationship between the abundance of the gut microbiota and the levels of inflammatory cytokines. As shown in Figure 5h, the abundance of *Alistipes* was negatively correlated with the levels of MSTN, TNF-α, and IL-6, the abundance of *norank_f_Lachnospiraceae* was negatively correlated with the levels of MSTN and TNF-α, the abundance of *Rikenella* was negatively correlated with the level of TNF-α, and the abundance of *unclassified_f_Lachnospiraceae* was negatively correlated with the level of MSTN. In addition, *Parasutterella* was positively associated with the three inflammatory factors. Therefore, the results of Spearman’s correlation analysis suggested a complex relationship between the gut microbiota and inflammatory cytokines. The treatments of R, W, and RW may lead to a change in gut microbiota environment and inflammatory status directly or indirectly, thus ultimately influencing skeletal muscle health through mutual interactions.

### 3.5. Results of Metabolomics Analysis

**Figure 6:**
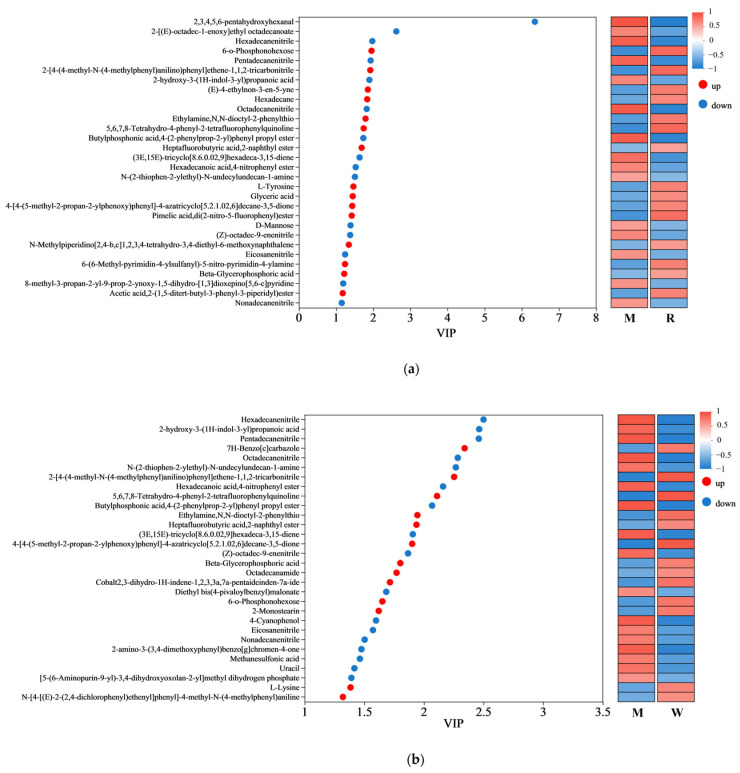

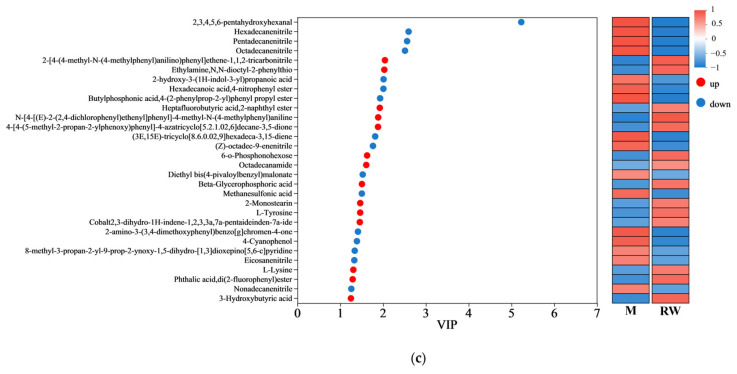
Variable importance in projection (VIP) scores. (**a**) The VIP analysis between the R group and the M group. (**b**) The VIP analysis between the W group and the M group. (**c**) The VIP analysis between the RW group and the M group. The red dots represent upregulated metabolites, while the blue dots represent downregulated metabolites. M was the model group, R was the rutin group, W was the whey protein group, RW was the rutin–whey protein nanoparticle group.

Non-targeted metabolomics was analyzed to compare the metabolic variations among the groups. As shown in the Supplementary Materials, the differential metabolites between groups were screened based on the OPLS-DA analysis (Table S1 and Figure S5), and are represented by volcano plots (Figure S6). Among the differential metabolites, the top 30 variables with VIP > 1 are shown in Figure 6. The regulation of metabolites by RW has both similarities and differences with the other two interventions. For example, in comparison with the mice in the M group, metabolites such as heptafluorobutyric acid,2-naphthyl ester, beta-glycerophosphoric acid, ethylamine, *N*,*N*-dioctyl-2-phenylthio were upregulated by all three treatment groups, while metabolites such as hexadecanenitrile, pentadecanenitrile, (Z)-octadec-9-enenitrile were downregulated by all three treatment groups. However, 3-hydroxybutyric acid was only upregulated by RW administration (Figure 6c). As evidence indicated, 3-hydroxybutyric acid exerted inhibitory effects on NOD-like receptor thermal protein domain-associated protein 3 (NLRP3) inflammasome activation, thereby attenuating the progression of NLRP3-mediated inflammatory pathologies [55]. Therefore, the difference in the metabolites may partially account for the better anti-inflammatory properties of RW, and such an effect of RW may not be the simple additive effect of R and W.

Amino acids are important components for the synthesis of many bioactive molecules involved in signal transduction, hormone production, reproduction, and muscle development [56]. Lysine is an essential amino acid that is abundant in muscle tissue [57]. Previous studies have shown that the skeletal muscle protein catabolism of fasting rats could be inhibited by oral administration of lysine, and muscle mass loss could also be suppressed by continuous feeding of the lysine-rich diet [58,59]. In addition, L-tyrosine could promote protein synthesis and metabolism, and contribute to the maintenance of muscle health and the promotion of physical recovery. A mouse model of nemaline myopathy exhibited severe muscle weakness and reduced mobility, but such symptoms could be alleviated by dietary L-tyrosine supplementation [60]. In this study, compared with the mice in the M group, the administration of R was found to upregulate the L-tyrosine level, and the administration of W was found to upregulate the L-lysine level, while the RW treatment was found to upregulate both (Figure 6a–c). Therefore, the above results showed that R, W, and RW might improve muscle mass and maintain muscle health in mice by regulating differential metabolites. The RW treatment could uniquely upregulate the 3-hydroxybutyric acid, L-tyrosine and L-lysine levels, which may explain its better anti-inflammatory properties and its ability to maintain muscle health.

**Figure 7:**
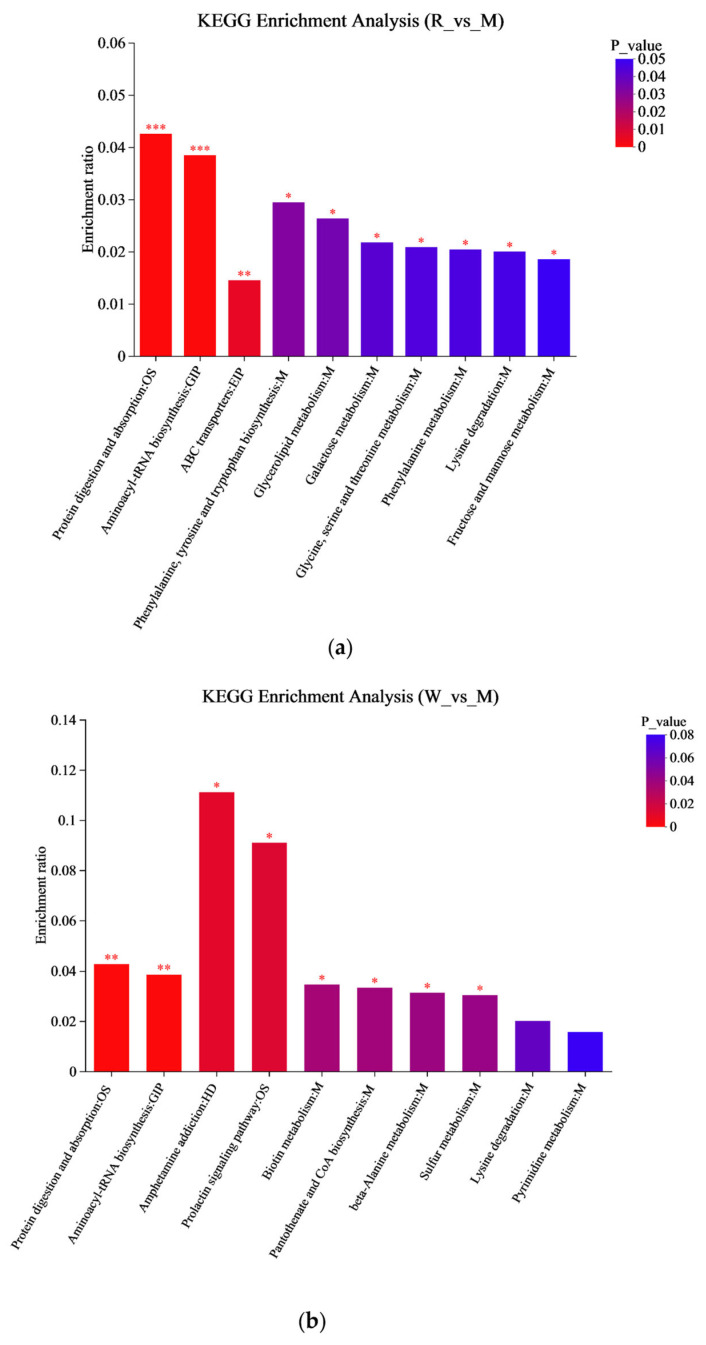

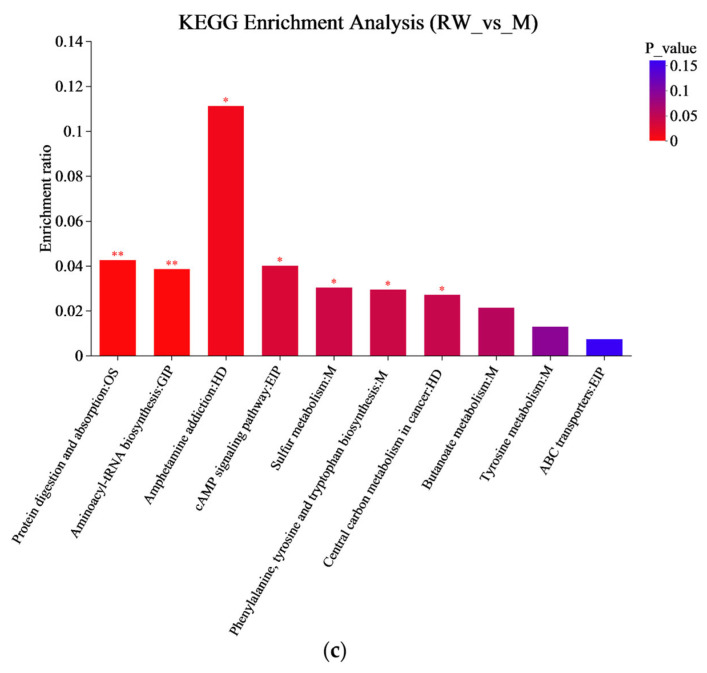
KEGG enrichment analysis. (**a**) The KEGG enrichment analysis between R and M. (**b**) The KEGG enrichment analysis between W and M. (**c**) The KEGG enrichment analysis graph between RW and M. M was the model group, R was the rutin group, W was the whey protein group, RW was the rutin–whey protein nanoparticle group. * *p* < 0.05, ** *p* < 0.01, *** *p* < 0.001.

A KEGG metabolic pathway enrichment analysis was carried out based on the metabolite differences between the groups, and the results are shown in Figure 7. The important pathways involved in the differential metabolites between the R and M groups were related to aminoacyl-tRNA biosynthesis, glycine, serine and threonine metabolism, phenylalanine, tyrosine and tryptophan biosynthesis, etc. (Figure 7a); the important pathways involved in the differential metabolites between the W and M groups were related to aminoacyl-tRNA biosynthesis, β-alanine metabolism, sulfur metabolism, etc. (Figure 7b), and the important pathways involved in the differential metabolites between the RW and M groups were related to aminoacyl-tRNA biosynthesis, sulfur metabolism, phenylalanine, tyrosine and tryptophan biosynthesis, etc. (Figure 7c). In the secondary classification of the KEGG pathway database, glycine, serine and threonine metabolism, as well as phenylalanine, tyrosine and tryptophan biosynthesis were classified under amino acid metabolism, sulfur metabolism was classified under energy metabolism and aminoacyl-tRNA biosynthesis was classified under translation. Therefore, R administration might regulate D-gal-induced metabolic dysfunction in mice through pathways such as amino acid metabolism and aminoacyl-tRNA biosynthesis; W treatment might regulate D-gal-induced metabolic dysfunction in mice through pathways like energy metabolism and aminoacyl-tRNA biosynthesis; and RW treatment might improve D-gal-induced metabolic abnormalities by regulating multiple metabolic pathways, including amino acid metabolism, energy metabolism, and aminoacyl-tRNA biosynthesis. These results were in accordance with previous reports. In previously published studies, royal jelly was proven to ameliorate D-gal-induced aging mainly through the regulation of metabolic pathways associated with energy metabolism, amino acid metabolism, lipid metabolism, and antioxidant pathways [61]. The anthocyanins from *Lycium ruthenicum* Murr. could restore the abnormal serum metabolomic profile of aging rats by significantly affecting amino-acid- and protein-related metabolic pathways [62]. It has also been found that theabrownin, as a polyphenol, could delay D-gal-induced aging in mice by regulating pathways such as lipid metabolism, purine metabolism, and aminoacyl-tRNA biosynthesis [41].

**Figure 8:**
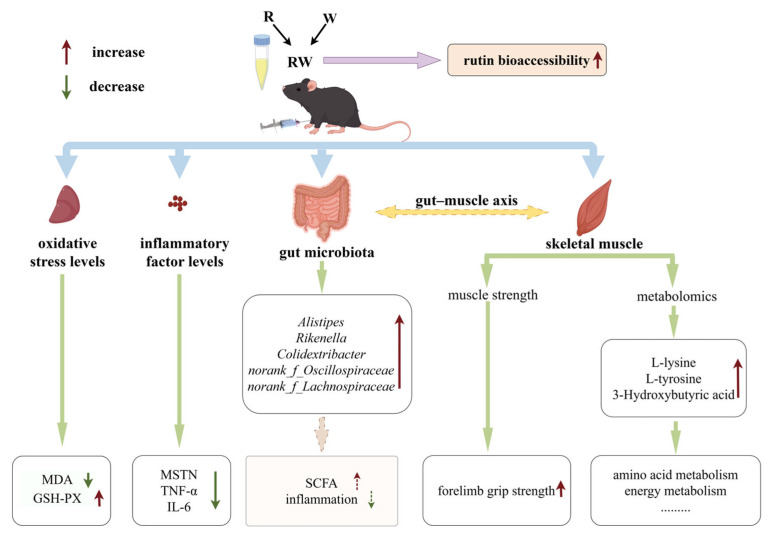
Potential effect and mechanism of RW on D-gal-induced skeletal muscle dysfunction (by Figdraw).

In this study, rutin was provided to mice at a dose of 100 mg/kg body weight, corresponding to a daily intake of approximately 486 mg for a 60 kg human [63]. Under this dosage, the administration of R significantly reduced the hepatic MDA level and the serum MSTN, IL-6, and TNF-α concentrations. However, it showed an insignificant effect on the skeletal muscle function in mice. When mice were administrated W, an improvement effect on hepatic antioxidant capacity and the serum inflammatory cytokine levels was also observed, while a significant influence on muscular strength was observed. Moreover, when rutin was combined with whey protein as nanoparticles, the bioaccessibility of rutin was markedly enhanced, which may lead to better in vivo effects. In our in vivo study, RW administration exhibited a significantly better effect than the individual effects of R or W on the regulation of hepatic GSH-PX activity and serum inflammatory cytokine levels. Additionally, the R, W, and RW treatments increased the relative abundance of some gut microbes that produce SCFAs or are involved in anti-inflammatory effects. Moreover, in addition to the metabolites commonly upregulated or downregulated across all three groups, the anti-inflammatory metabolite 3-hydroxybutyric acid was upregulated by RW administration, and both L-tyrosine and L-lysine were upregulated by the RW treatment. Therefore, compared with R or W, RW administration exhibited a potentially more comprehensive effect on muscle function protection (Figure 8).

## 4. Conclusions

In this research, rutin and whey protein were developed as nanoparticles with a uniform particle size, high encapsulation efficiency, high loading capacity, and enhanced bioaccessibility. This in vivo study indicated that R, W and RW could enhance hepatic antioxidative capacity and regulate inflammation levels in D-gal-induced mice, while W and RW could also increase muscle strength. Among these, the RW treatment exhibited significantly better effects on elevating hepatic GSH-PX activity and lowering the serum MSTN, TNF-α, IL-6 levels than those of the individual effect of R or W administration. The mechanism of R, W, and RW administration on the prevention of skeletal muscle dysfunction may be achieved through the modulation of the gut microbiota and metabolic profiles.

## Data Availability

The original contributions presented in this study are included in this article; further inquiries can be directed to the corresponding author.

## Supplementary Materials

The following supporting information can be downloaded at: https://www.mdpi.com/article/10.3390/nu17101734/s1. The specific testing methods for skeletal muscle movement ability and grip strength test; fresh manure collection methods; the data analysis method of gut microbiota analysis; detailed procedures, conditions and data analysis for metabolomics analysis; Figure S1: A graph of body weight changes in mice; Figure S2: Effects of R, W and RW treatments on muscle histopathology; Figure S3: Venn diagram of OTUs; Figure S4: Effects of R, W and RW treatments on the Firmicutes/Bacteroidota ratio; Figure S5: OPLS-DA score plot; Figure S6: Volcanic map of differential metabolites between groups; Table S1: R^2^X, R^2^Y, and Q^2^ values of OPLS-DA models.
