# Supplementary material for: Rutin–Whey Protein Nanoparticles Inhibit D-Galactose-Induced Skeletal Muscle Dysfunction by Modulating Gut Microbiota and Metabolic Pathways
Source: Nutrients. 2025 May 20;17(10):1734. doi: 10.3390/nu17101734 (PMC12113907; doi:10.3390/nu17101734)
Supplement: Supplementary file 1 [file nutrients-17-01734-s001.zip › nutrients-3605787 Supplementary Material .pdf]

Supplementary Materials

Skeletal muscle movement ability. Following acclimatization to the treadmill apparatus, mice were subjected to constant-speed running at 14.0 m/min until exhaustion. The operational definition of exhaustion was established as 10 consecutive seconds of continuous contact with the electrical stimulus grid without demonstrating forward locomotion. The movement distance under the exhaustion state was recorded.

Grip strength test. Briefly, the forelimbs of the mice were naturally gripped on the horizontally mounted gripping net, and a force parallel to the net was applied to the mice tail until the forelimbs dislodged from the net. Results displayed on the device screen were recorded as the forelimb grip force. Before the formal test, the mice were acclimatized to the test for two days, and the test was repeated five times continuously to calculate average results.

Fresh manure collection methods. One hand grasps the mouse, while the other hand gently massages the abdomen to induce the excretion of fresh feces into a sterile centrifuge tube.

Gut microbiota analysis. The analysis of the gut microbiota in feces was carried out using the Majorbio Cloud platform (https://cloud.majorbio.com). Based on the OTUs information, alpha diversity indices were calculated with Mothur (version 1.30.2). Principal coordinates analysis (PCoA) was employed to evaluate the similarity of microbial communities across different groups. Statistically significant differences in relative abundances between different groups were determined using the Kruskal–Wallis H test, with significant differences at $p < 0.05$. Heatmap analysis was performed based on Spearman's correlation coefficients to visualize the relationships between dominant genera and inflammatory markers.

Metabolomics analysis. Chromatographic separation was performed on a DB-5MS capillary column (40 m×0.25 mm×0.25 μm) using ultra-high purity helium (99.999%) as carrier gas maintained at a constant flow rate of 1 mL/min. The GC oven temperature program was initialized at 60 °C for 30 seconds, followed by a linear ramp of 8 °C/min to 310 °C, then held for 6 minutes. The sample injection volume was 1 μL and introduced in splitting mode (15:1) with the inlet temperature of 260 °C. The mass spectrometry acquisition parameters include: 230 °C for the ion source temperature and 150 °C for the quadrupole temperature. The full scan mode was selected, and the quality scanning range was 50-500 m/z, with the scanning frequency as 3.2 scan/s. Metabolomics data analysis was conducted on the Majorbio cloud platform. The orthogonal partial least squares discriminant analysis (OPLS-DA) of differential metabolites were conducted with the R packages (Version 1.6.2). Differential metabolites were filtered based on variable importance in projection (VIP) values exceeding 1.0 and p value less than 0.05. Functional annotation of metabolic pathways was conducted by mapping metabolites to the KEGG pathway database (https://www.kegg.jp/kegg/pathway.html). Pathway enrichment analysis was executed using the SciPy scientific computing library (Version 1.0.0) in Python.

Table S1 R^2X, R^2Y, and Q^2 values of OPLS-DA models.

model	R^2X(cum)	R^2Y(cum)	Q^2(cum)
M vs C	0.536	0.998	0.873
R vs M	0.349	0.995	0.89
W vs M	0.386	0.996	0.912
RW vs M	0.428	0.991	0.926

Note: C was the control group, M was the model group, R was the rutin group, W was the whey protein group, RW was the rutin–whey protein nanoparticle group.

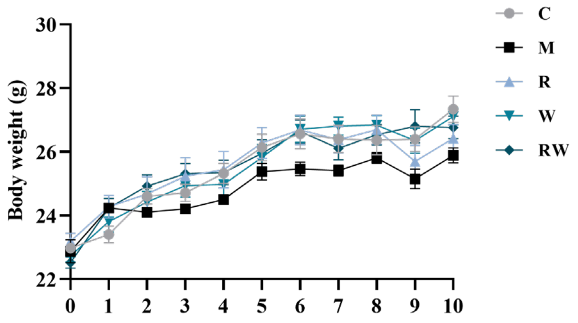

Figure S1. A graph of body weight changes in mice. C was the control group, M was the model group, R was the rutin group, W was the whey protein group, RW was the rutin–whey protein nanoparticle group.

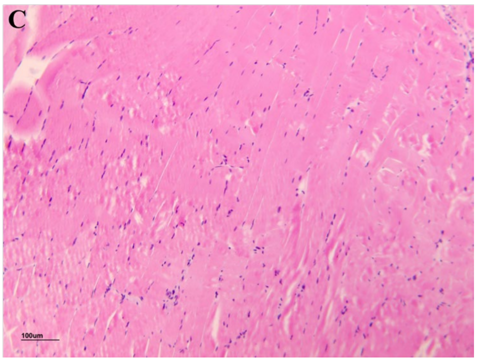

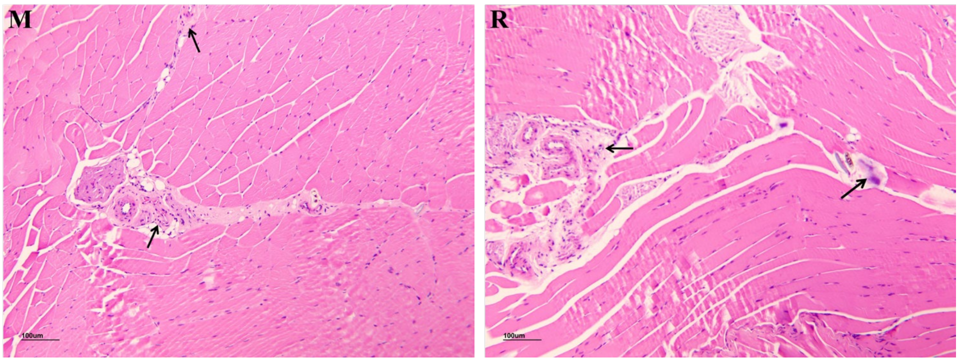

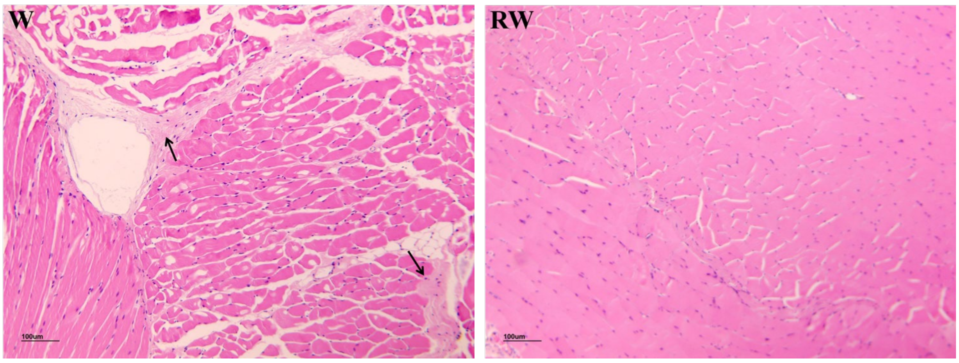

Figure S2. Effects of R, W and RW treatments on muscle histopathology. Scale bar in the figure represented 100 μm. C was the control group, M was the model group, R was the rutin group, W was the whey protein group, RW was the rutin–whey protein nanoparticle group. Arrows point to inflammatory cell infiltration.

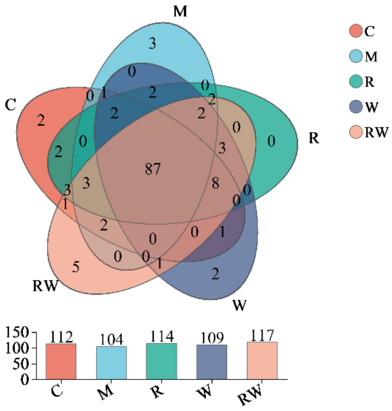

Figure S3. Venn diagram of OTUs. C was the control group, M was the model group, R was the rutin group, W was the whey protein group, RW was the rutin–whey protein nanoparticle group.

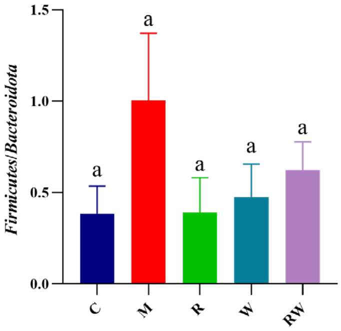

Figure S4. Effects of R, W and RW treatments on the *Firmicutes / Bacteroidota* ratio. C was the control group, M was the model group, R was the rutin group, W was the whey protein group, RW was the rutin–whey protein nanoparticle group.

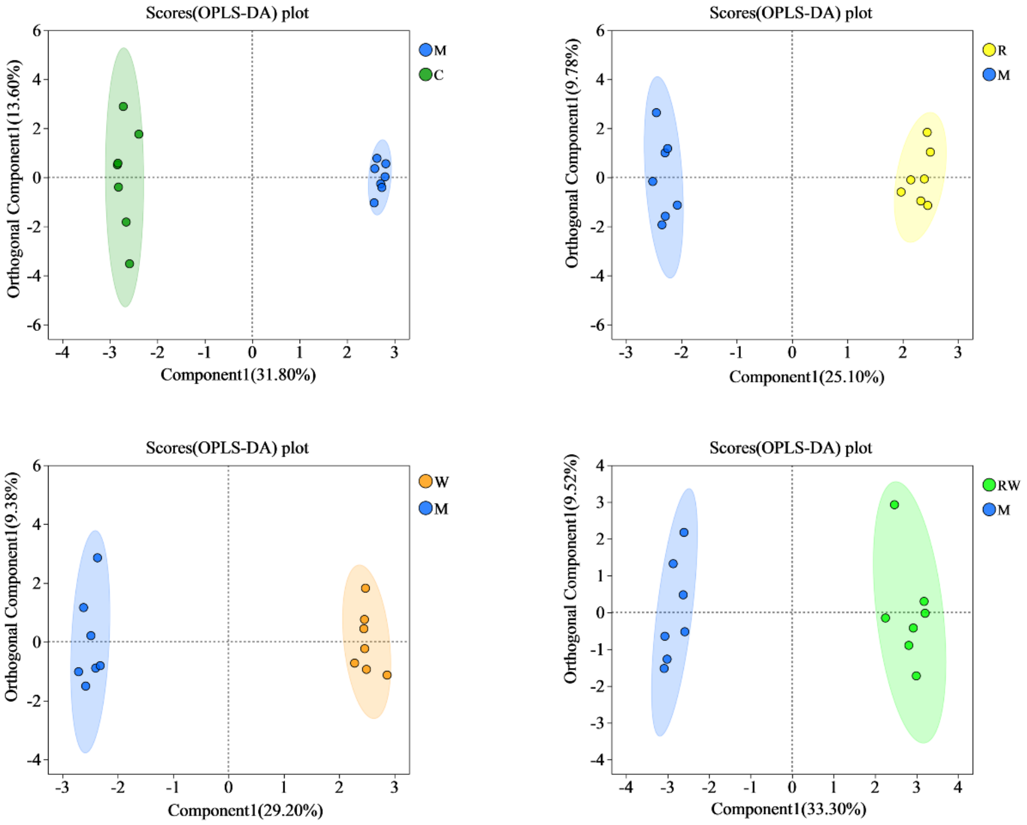

Figure S5. OPLS-DA score plot. C was the control group, M was the model group, R was the rutin group, W was the whey protein group, RW was the rutin–whey protein nanoparticle group.

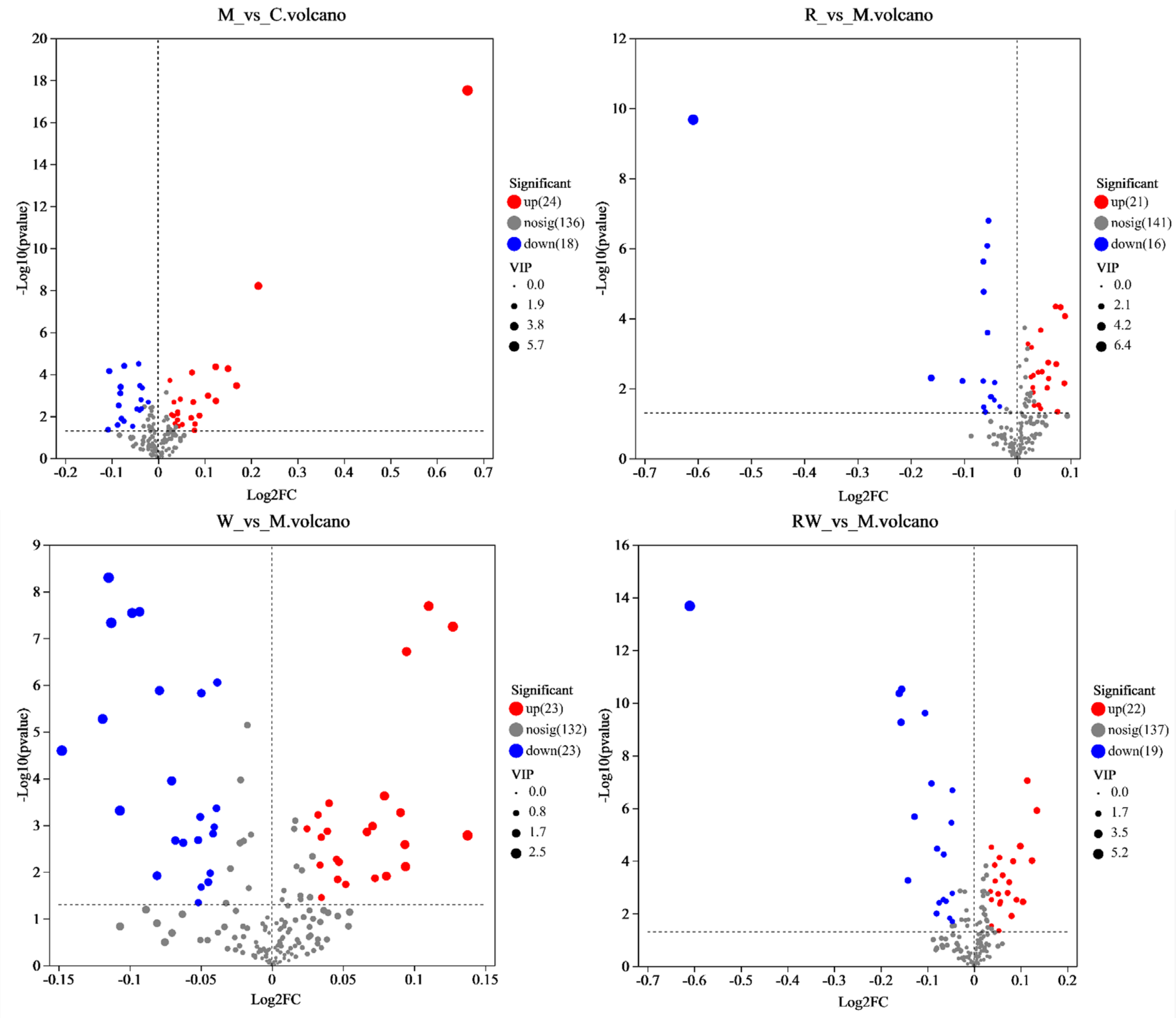

Figure S6. Volcanic map of differential metabolites between groups. Red dots represent up-regulated metabolites and blue dots represent down-regulated metabolites. C was the control group, M was the model group, R was the rutin group, W was the whey protein group, RW was the rutin–whey protein nanoparticle group.
